# Compulsory Military Service

**Published:** 1915-04

**Authors:** 


### Compulsory Military Service.

Without subscribing to the views of military alarmists, certain facts must be admitted:

1. Owing to the practical development of science, neither this country nor any other in the world, can depend upon geographic remoteness, for protection. This depends, not so much upon improved facilities for transportation of military forces and equipment as upon the rapid transmission of news. Only actual, long continued reasons for disagreement can precipitate war when those reasons are known weeks afterward but, with practically instantaneous communication, a quarrel may readily develop into war.

2. War does not depend upon logic but upon preconceived intentions. Neither does it take two to make a quarrel. Without considering any of the potential causes of war as adequate, it must be recognized that war will occur if, with or without excuse, any nation—or rather any small body of military authorities—desires it. No human intelligence can prophesy accurately.

3. War with any major power will be on a large scale, requiring both men and equipment approximately equal to whatever force can be mustered by any single nation or conceivable group of allies, with due regard to the length of natural defense lines.

4. Under existing circumstances, no nation can depend for defense upon a professional army, supported on an economic basis like a fire or police department but must depend upon something approaching universal, compulsory conscription. While, ultimately, the assembling of untrained men, actuated by patriotism may be successful, such a policy will involve great delay, and enormous preliminary losses, not to mention the unpleasant detail of the application of the rules of war to "snipers."

It may not be out of place, at this point, to dispute the statement so often repeated, as an argument for non-preparedness, that the Revolution was fought by untrained men. Without dilating on the difference between conditions at that time and the present, which refute any complaisant analogy, and without in any way detracting from the credit given to Steuben, Lafayette and other European officers for the training which they gave the Continental army, it may be confidently stated that, with reference to the conditions existing, the American forces were far from being untrained. Probably the majority of the men had had previous experience in regularly organized military bodies, many in actual warfare. Almost all were familiar with the use of firearms. Bodies of men were regularly drilled in expectation of the Revolution, and details of mobilization were worked out to a degree of perfection which is surprising when we consider the lack of facilities for transmission of news. Troops were raised and officers commissioned in Vermont, in 1774 and much earlier than this in New England, with definite reference to a Revolution; indeed such a possibility was noted by Sir John Evelyn of a board of correspondence with colonial governors, almost a century earlier and a definite policy of conciliation was adopted so far as the American governors were concerned.

It may be good poetry to ascribe the success of the Revolution to the spontaneous patriotism of farmers, merchants, and mechanics, rising to defend their liberties and it would be going too far to claim a regularly organized military body for the American colonies but the fact is that, almost to a man, they were skilled, according to the standards of their time, in the use of weapons, that about 20 per cent. of them and perhaps more, had had pretty thorough military training and probably 10 per cent. had had actual experience in war. Even general matters of organization, mobilization, provisioning, etc., had been arranged so far as was practicable and, with due regard to the numbers involved, the range of possible operation and the inevitable dependence upon local action which the lack of scientific and material development entailed even upon regular troops, the strategic preparedness was, relatively, on a higher plane than at the beginning of the Civil or the Spanish-American war.

Approximately 1 per cent. of any population consists of males of a given age usually assigned for compulsory military service. In other words, if the United States, adopted compulsory military service for one year, after the completion of education in the formal sense and before entrance upon lifework, the standing army of militia would consist of about one million. In a few years, an adequate defensive force, comparatively fresh from military training, would be available, provided occasional assemblies were inforced, details of mobilization by units, were arranged, and equipment were at hand. It should be borne in mind that niceties of drill, manual of arms, and even accuracy of fire, do not count for much in actual warfare. Rapidity of assembling into military units, habits of obedience to military discipline, physical stamina and practical knowledge of personal hygiene are the main factors.

The experience of European countries leads one to question whether compulsory military service is the economic drain which appears so conspicuously. The inculcation of discipline, both to command and to obey; the physical training, the checking of inebriety and other bad habits, the acquisition of the ability to get along with one's associates, without undue friction, the habituation to regularity of life and to occasional emergency demands to break this regularity, the practical and didactic instruction in sanitary and hygienic matters, seem in the course of the average life, to render the citizen more amenable to law, more industrious, more efficient, and thus to outweigh the direct expense and the subtraction of a year or more from industrial occupation. Imagine, for example the tremendous economic gain that would accrue to this country if its workers generally realized the necessity of doing a thing to accomplish the desired result, harmoniously and efficiently, without regard to preconceived notions of personal convenience, appropriate hours, and conflicting social engagements.

For this country, conditions are such, that it is doubtful whether it would be necessary to have military service at the expense of a year's industrial usefulness. Preliminary training could be combined with school work. It has indeed, already been thus combined in numerous instances and almost without exception, to the satisfaction of both the students, the school authorities and even to the military trainers—with due regard to the degree to be expected. If, beyond such preliminary training, a schedule of three evenings a week, one afternoon a week, and two months of summer encampment, were adopted, and inforced for a year, there would be no considerable interference with the first year of industrial life of the average youth and while the result would not be entirely satisfactory from the military standpoint, it would do away with the delay of recruiting camps and would, within a very few years, secure us an army of fair efficiency and adequate size, with each unit assigned to its proper place, and capable of being uniformed and armed within a day or two—granting of course that proper supplies were in readiness. Such an army could be stationed to meet an invading force—for an invading force of large dimensions cannot conceivably enter the country by surprise—and within a few months would be equal in efficiency to any except the most highly trained force of veterans.

The necessary expense might be considered as an insurance of peace and we feel certain that the economic increase in the country's material welfare would much more than compensate for the expense, for there is no question but that the general feeling of security would be greater than on a merely sentimental protection against war. But, even if no such insurance were necessary, we feel that such a military establishment would pay for itself in other ways, for instance, the lessening of criminality which is an expensive luxury, the greater aggregate efficiency and health of the men of the country, even in the indirect results of the patriotism and fraternal association which would develop. For our own profession and similarly for engineers, of various kinds, for those interested in matters of transportation, of preparing and distributing all sorts of equipment, the service would be especially valuable. Let us take merely one point, applying to our own profession: the experience, with the responsibility involved and under proper guidance, of examining large numbers of men as to their physical condition, would be worth the time spent to the average physician.

